# Surgical Outcomes in Patients Exhibiting Muscular Weakness Postadolescence Due to Tight Filum Terminale

**DOI:** 10.7759/cureus.64080

**Published:** 2024-07-08

**Authors:** Takaki Kitamura, Yasuaki Murata, Tomonori Shigemura, Yohei Yamamoto

**Affiliations:** 1 Department of Orthopedic Surgery, Graduate School of Medicine, Chiba University, Chiba, JPN; 2 Department of Orthopedic Surgery, Asahi General Hospital, Asahi, JPN; 3 Department of Orthopedics, Teikyo University Chiba Medical Center, Ichihara, JPN

**Keywords:** postadolescent, bowel and bladder dysfunction, muscle weakness, untethering, tight filum terminale

## Abstract

Introduction: Tight filum terminale is a neurological condition marked by various symptoms, including muscle weakness. There is a notable lack of literature addressing muscle weakness, particularly in cases emerging during adolescence and beyond. The diagnosis is challenging due to a lack of radiological abnormalities, and the literature on its treatment, especially untethering, in adults is limited. This study aims to evaluate the effectiveness of untethering in improving muscle weakness and other symptoms in postadolescent patients diagnosed with tight filum terminale.

Methods: A retrospective analysis was conducted on seven postadolescent patients diagnosed with tight filum terminale and presenting muscle weakness who underwent untethering at our institution between January 2018 and August 2022. Patients were monitored for muscle strength improvement, lumbar and lower extremity pain, and bowel and bladder dysfunction (BBD) after untethering.

Results: Muscle weakness improved in all cases after untethering, with a mean duration of 9.1 weeks for the improvement. Patients unable to walk independently regained mobility in an average of 22.3 weeks. Lumbar and lower limb pain improved in all cases within an average of 8.1 weeks, while BBD improved in six of the seven cases within an average of 1.9 weeks.

Conclusions: Our findings suggest that untethering is an effective surgical intervention for postadolescent patients diagnosed with tight filum terminale and presenting muscle weakness.

## Introduction

Tight filum terminale is a condition resulting from excessive traction on the spinal cord due to the filum terminale [1]. It is regarded as a tethered cord syndrome variant lacking imaging abnormalities such as low conus or lipoma, differentiating it from other forms. Due to the absence of imaging abnormalities, it is sometimes called occult tethered cord syndrome [2]. Tight filum terminale is characterized by neurological symptoms, urinary and bowel dysfunction, and orthopedic manifestations [1,3-5]. It can also affect the upper limbs and cause diffuse lower limb muscle weakness, complicating diagnosis based on physical examination [6]. Untethering is a surgical treatment for this condition [5,7].

Reported differences exist in the symptoms of tight filum terminale between pediatric and adult-onset cases [8,9]. Similarly, the age of onset may influence the symptoms and treatment outcomes of tight filum terminale. While numerous studies have documented the outcomes of untethering surgery for tight filum terminale in pediatric patients, data on the outcomes of untethering surgery for adult patients with tight filum terminale are scarce, and its efficacy remains unclear [3,5,10-17]. The literature indicates significant improvement in lower back and leg pain following untethering surgery, but there is a lack of documentation on muscle weakness, particularly in cases diagnosed after adolescence [5].

This study evaluates the effects of untethering surgery on patients who developed tight filum terminale with muscle weakness after adolescence. The aim of this research is to clarify the efficacy of untethering treatment for tight filum terminale in adults, with a specific focus on improving muscle weakness.

## Materials and methods

**Figure 1:**
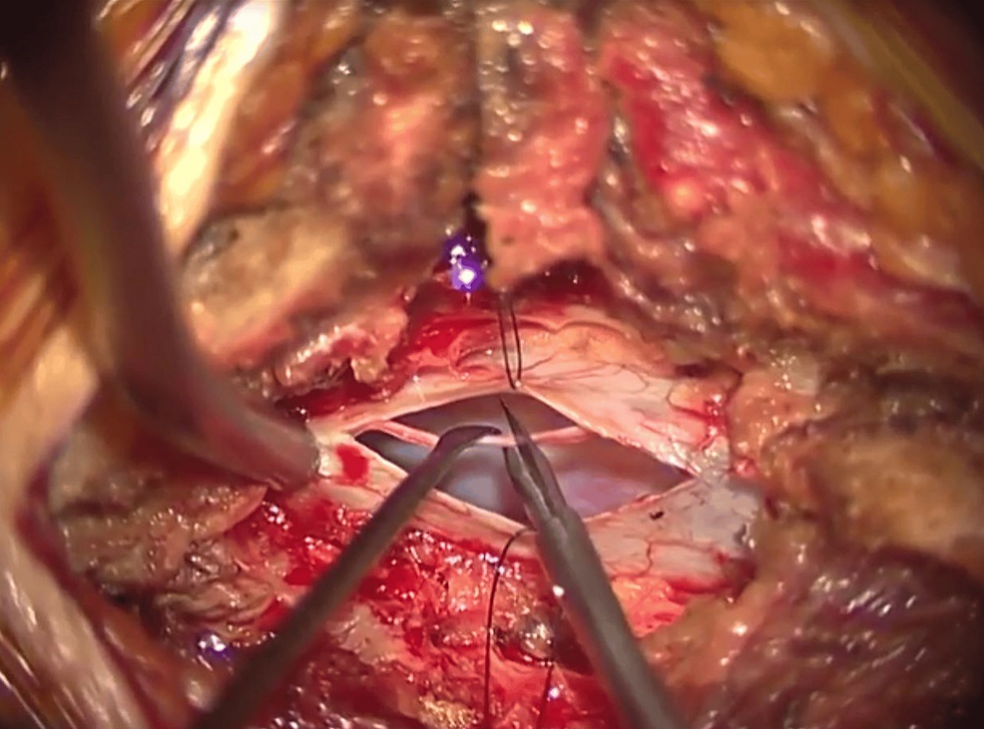
Untethering of the filum terminale A surgical incision of the dura mater at the L5/S1 intervertebral level was performed, identifying the filum terminale using somatosensory evoked potential monitoring, and further confirmed that there was no muscle contraction in the lower limbs upon electrical stimulation before proceeding with untethering

From January 2018 to August 2022, we conducted a retrospective analysis of cases presented at our institution diagnosed with tight filum terminale employing the Komagata criteria [3]. The Komagata criteria encompass five distinct components utilized for diagnostic evaluations. These include (1) the presence of back or lower limb pain; (2) limited trunk flexion, characterized by a finger-to-floor distance of at least 20 cm; (3) altered bladder or bowel function, such as urinating eight or more times daily; (4) neurological symptoms that do not correlate with findings on imaging studies; and (5) a positive response to the tight filum terminale provocation test. The tight filum terminale provocation test is an assessment where symptoms are induced through both trunk anteflexion and excessive neck flexion. The elicited symptoms resolve upon the relaxation of the neck flexion, confirming a positive test result. There were 15 cases diagnosed with tight filum terminale and underwent surgery. Among them, seven cases showing muscle weakness were the subjects of this study. There were three male and four female participants, with an average age of 29.0 years (range 17-46 years). All surgical interventions constituted untethering. The surgical methodology commenced with an incision into the dura mater at the L5/S1 intervertebral junction. Subsequently, identification of the filum terminale was facilitated through somatosensory evoked potential monitoring. Ultimately, meticulous sectioning of the filum terminale was performed employing microscissors (Figure 1).

Muscular debilitation was characterized by a manual muscle test (MMT) score of 4 or lower in any lower extremity muscle, whereas muscle improvement was denoted as an increment of at least a 1-point MMT score. The primary outcome measures encompassed muscle strength improvement, the timeframe from acute exacerbation to surgical intervention, and the interval from surgery to progress. The secondary outcome measures included the improvement in lumbosacral and lower extremity discomfort, as well as bowel and bladder dysfunction (BBD) and the corresponding temporal patterns of improvement. BBD was delineated in accordance with the guidelines set forth by the International Children's Continence Society (ICCS) [18]. In patients exhibiting a tight filum terminale, one of the prevalent symptoms of BBD is urinary instability, which, according to the ICCS guidelines, is characterized by a urinary frequency exceeding eight times per day. Recovery from urinary instability, in this context, is defined as a reduction of urinary frequency to fewer than five times per day. The examination for frequent urination includes blood tests, urine tests, physical examination, and consultation with a urologist to exclude frequent urination caused by other diseases.

## Results

There were three cases of inability to walk independently. The average duration from acute exacerbation to surgery was 19.1 weeks, with an average observation period of 18 months. Muscle weakness improved in all cases after untethering. The average duration to achieve at least a 1-point MMT improvement was 9.1 weeks. The average time for cases unable to walk independently to regain independent walking was 22.3 weeks (Table 1).

**Table 1:** Patient data on muscle strength and walking ability before and after surgery ^a^The timeframe from acute exacerbation to surgery ^b^The duration of at least one-point MMT score recovery ^c^The duration necessary for both MMTs to attain a 5-point score, facilitating an unassisted walk MMT: manual muscle test

Patient	Age	Sex	Observation period (month)	Time to surgery (week)^a^	Presurgery MMT	Presurgery independent walk	Postsurgery MMT	Duration of motor recovery (week)^b^	Duration of full motor recovery (week)^c^
1	26	M	27	15.9	4	Yes	5	0.3	0.3
2	37	M	14	10.9	4	Yes	5	12.6	12.6
3	42	F	9	30.0	4	Yes	5	12.3	12.3
4	18	F	9	5.4	4	Yes	5	3.6	3.6
5	17	F	36	39.4	3	No	5	6.4	10.3
6	17	F	7	12.6	2	No	5	17.3	31.3
7	46	M	22	19.7	1	No	5 (right), 4 (left)	4.0	25.3

All cases experienced lumbar and lower limb pain, which improved in an average of 8.1 weeks. BBD was observed in six of seven cases, with all cases presenting with urinary frequency symptoms. Urinary frequency improved in an average of 1.9 weeks (Table 2).

**Table 2:** Outcomes and recovery durations for LBP and BBD before and after surgery LBP: low back pain; BBD: bowel and bladder dysfunction; N/A: not applicable

Patient	Presurgery LBP	Postsurgery LBP	Duration of LBP recovery (week)	Presurgery BBD	Postsurgery BBD	Duration of BBD recovery (week)
1	Yes	No	0.3	Yes	No	2.4
2	Yes	No	12.6	No	No	N/A
3	Yes	No	11.6	Yes	No	2.3
4	Yes	No	15.6	Yes	No	1.1
5	Yes	No	10.3	Yes	No	1.1
6	Yes	No	5.6	Yes	No	1.4
7	Yes	No	1.0	Yes	No	2.7

## Discussion

Tight filum terminale, also referred to as occult tethered cord syndrome, is considered one of the types of tethered cord syndromes. It is distinguished from tethered cord syndrome by the absence of radiological abnormalities, such as low conus or lipomas [19]. In pediatric cases, it is thought to occur due to the difference in growth between the spinal cord and vertebrae, causing the spinal cord to be tractioned. On the other hand, in adult cases, it is believed that patients with a mildly tractioned spinal cord may develop symptoms due to some external factors related to spinal movement, often resulting in an acute onset [4]. The symptoms of tethered cord syndrome are diverse, including BBD, lower limb dysfunction, lumbar and lower limb pain, and sensory disturbances, making diagnosis challenging [5]. Untethering is a common surgical treatment for symptomatic tethered cord syndromes and has been reported in many pediatric cases [5,10-13,19,20]. However, its indications for occult tethered cord syndrome remain controversial. Moreover, there are very few reports on the treatment outcomes of tight filum terminale in adult-onset cases [5].

Untethering for tight filum terminale improves muscle weakness [5]. Muscle weakness is observed in 9%-40% of tethered cord syndrome patients, which is relatively rare compared to the prevalence of lumbar and lower limb pain or BBD. Surgical treatment with untethering has been reported to show motor improvement in 25%-100% of patients [5]. In this study, all cases, including those with severe muscle weakness rendering them unable to walk independently, exhibited improved muscle weakness. Most previous studies have included pediatric cases, and the timing of acute exacerbation has not been clearly reported, resulting in few reports on the duration from acute exacerbation to surgery [5]. In this study, surgery was performed on average 19 weeks after acute exacerbation, with all cases diagnosed and treated within one year of worsening symptoms. The duration for muscle strength to improve by at least 1 MMT score varied widely, from 0.3 to 17.3 weeks, with no clear relationship to the timing of surgical intervention. On average, it took 22.2 weeks for nonambulatory cases to regain the ability to walk independently, suggesting that earlier surgical intervention before severe impairment may enable a more rapid return to societal participation. In a chronic continuous spinal cord traction model in cats, it has been reported that symptoms gradually improve over time, suggesting that adaptive mechanisms may be at work in the spinal cord depending on the degree and duration of spinal cord tethering [5]. This implies that conservative treatment may improve symptoms in some cases. However, in pediatric cases, there have been reports of greater surgical improvement in cases with more than two of the four categories of symptoms (neurologic, urologic, orthopedic, and dermatologic) compared to cases with only one symptom [5]. In this study, all cases exhibited muscle weakness and lumbar and lower limb pain, and six cases also had BBD, suggesting no contradiction in surgical indications. It is crucial to perform surgery before the spinal cord sustains irreversible damage.

Lumbar and lower extremity pain is noted in 25%-69% of patients, with complete amelioration accomplished via surgical intervention [5]. In this study, we witnessed enhancements in all subjects, exhibiting a broad spectrum of improvement durations between 0.3 and 15.6 weeks. Lumbar and lower extremity pain represents a symptom anticipated to improve following surgery. Nevertheless, there exist accounts of postoperative pain recurrence, necessitating the continuation of postoperative monitoring.

BBD manifests in 40%-100% of patients, with postoperative amelioration observed in over 60% of cases [5]. This symptom is predominantly documented in pediatric instances. In the present study, urinary frequency symptoms were discerned in six out of seven subjects, with an absence of bowel dysfunction. Furthermore, the postoperative enhancement interval ranged from 1.1 to 2.7 weeks, suggesting the potential for expeditious improvement of urinary frequency symptoms in adult-onset cases.

Tight filum terminale poses a formidable diagnostic challenge stemming from the lack of discernible anomalies in imaging findings [1]. The scarcity of reports in adult cases, particularly those involving muscular debilitation, further complicates matters [3,5,10,11,14-17,21]. This investigation, too, comprises a limited cohort. A more extensive accumulation of cases is essential for ascertaining suitable candidates and optimal timing for surgical intervention. Furthermore, instances of recurring lumbar and lower extremity discomfort, coupled with BBD, necessitate persistent long-term postoperative monitoring. In this particular study, follow-up was constrained to a mere 18-month average, underscoring the need for ongoing evaluation of patient outcomes.

## Conclusions

Untethering is efficacious for patients experiencing postadolescent tight filum terminale concurrent with muscular insufficiency. The mean time required to attain a minimum of a 1-point enhancement in MMT was 9.1 weeks. While the results are encouraging, a more extensive accumulation of cases and long-term follow-up studies are needed to confirm these findings and to further refine the treatment strategies for this condition.
